# Clinical characteristics and predictors of mechanical ventilation in patients with COVID-19 hospitalized in Southern Brazil

**DOI:** 10.5935/0103-507X.20200082

**Published:** 2020

**Authors:** Gisele Alsina Nader Bastos, Aline Zimmermann de Azambuja, Carisi Anne Polanczyk, Débora Dalmas Gräf, Isabelle Weschenfelder Zorzo, Juçara Gasparetto Maccari, Lúcia Schnor Haygert, Luiz Antônio Nasi, Marcelo Basso Gazzana, Marina Bessel, Paulo Márcio Pitrez, Roselaine Pinheiro de Oliveira, Marcelo Comerlato Scotta

**Affiliations:** 1 Hospital Moinhos de Vento - Porto Alegre (RS), Brazil.; 2 Universidade Federal de Ciências da Saúde de Porto Alegre - Porto Alegre (RS), Brazil.; 3 Hospital de Clínicas de Porto Alegre, Universidade Federal do Rio Grande do Sul - Porto Alegre (RS), Brazil.; 4 Universidade Federal do Rio Grande do Sul - Porto Alegre (RS), Brazil.; 5 Pontifícia Universidade Católica do Rio Grande do Sul - Porto Alegre (RS), Brazil.

**Keywords:** COVID-19, Coronavirus infections, SARS-CoV-2, Cohort studies, Risk factors, Respiration, artificial, Pandemics, COVID-19, Infecções por coronavírus, SARS-CoV-2, Estudos de coorte, Fatores de risco, Respiração artificial, Pandemia

## Abstract

**Objective:**

This study aims to describe the clinical characteristics and predictors of mechanical ventilation of adult inpatients with COVID-19 in a single center.

**Methods:**

A retrospective cohort study was performed and included adult inpatients hospitalized from March 17th to May 3rd, 2020, who were diagnosed with SARS-CoV-2 infection. Clinical and demographic characteristics were extracted from electronic medical records.

**Results:**

Overall, 88 consecutive patients were included in this study. The median age of the patients was 63 years (IQR 49 - 71); 59 (67%) were male, 65 (86%) had a college degree and 67 (76%) had at least one comorbidity. Twenty-nine (33%) patients were admitted to the intensive care unit, 18 (20%) patients needed mechanical ventilation, and 9 (10.2%) died during hospitalization. The median length of stay in the intensive care unit and the median duration of mechanical ventilation was 23 and 29.5 days, respectively. An age ≥ 65 years was an independent risk factor for mechanical ventilation (OR 8.4 95%CI 1.3 - 55.6 p = 0.02).

**Conclusion:**

Our findings describe the first wave of Brazilian patients hospitalized for COVID-19. Age was the strongest predictor of respiratory insufficiency and the need for mechanical ventilation in our population.

## Abstract (pt)

**Objetivo:**

Descrever as características clínicas e os preditores de ventilação mecânica em pacientes adultos internados com COVID-19.

**Métodos:**

Conduziu-se um estudo de coorte retrospectiva com inclusão de pacientes hospitalizados entre 17 de março e 3 de maio de 2020, que tiveram o diagnóstico de infecção pelo SARS-CoV-2. As características clínicas e demográficas foram extraídas de registros em prontuário eletrônico.

**Resultados:**

Incluíram-se no estudo 88 pacientes consecutivos. A mediana da idade dos pacientes foi de 63 anos (IQR: 49 - 71); 59 (67%) pacientes eram do sexo masculino, 65 (86%) tinham educação universitária e 67 (76%) tinham, no mínimo, uma comorbidade. Dentre eles, 29 (33%) pacientes foram admitidos à unidade de terapia intensiva, 18 (20%) necessitaram de ventilação mecânica e nove (10,2%) morreram durante a hospitalização. O tempo mediano de permanência na unidade de terapia intensiva e o tempo mediano de ventilação mecânica foram, respectivamente, de 23 e 29,5 dias. Idade acima ou igual a 65 anos foi fator de risco independente para ventilação mecânica (RC: 8,4; IC95% de 1,3 - 55,6; valor de p = 0,02).

**Conclusão:**

Nossos achados descrevem a primeira onda de pacientes brasileiros hospitalizados por COVID-19. Em nossa população, idade foi o maior preditor de insuficiência respiratória e necessidade de ventilação mecânica.

## INTRODUCTION

Since the first report in Wuhan, China in December of 2019, the outbreak of a new strain of coronavirus, named severe acute respiratory syndrome coronavirus type-2 (SARS-CoV-2), has spread globally. The disease caused by this new virus, Coronavirus Disease 19 (COVID-19), leads to acute respiratory distress syndrome (ARDS) in approximately 5% of diagnosed patients. On March 11, 2020, the World Health Organization (WHO) declared it as a pandemic, and as of September 20, 2020, 30,675,675 cases of COVID-19 had been diagnosed worldwide, with 954,417 related deaths.^(1,2)^

Latin America was affected later by the pandemic, and limited data are available regarding the characteristics and clinical course of the disease in this continent. The first diagnosed case of COVID-19 in Brazil occurred on February 26, 2020 in the city of São Paulo (SP). In the state of Rio Grande do Sul, the Southernmost Brazilian state, the first patient with COVID-19 was identified on March 10 in Porto Alegre, the State’s capital. As SARS-CoV-2 infection was introduced in Brazil from individuals traveling overseas, most of the patients diagnosed during the first weeks of the pandemic in Brazil were subjects with high socioeconomic status and were admitted to private hospitals. On September 20, 2020, Brazil reported 4,528,240 COVID-19 confirmed cases and 136,532 COVID-19-related deaths, leading to a case fatality rate of 3%. On the same day, the state of Rio Grande do Sul reported 174,140 COVID-19 confirmed cases and 4.371 COVID-19-related deaths, leading to a case fatality rate of 2.5%.^(3,4)^

The COVID-19 pandemic has been associated with an increase in the prevalence of complications among patients of older age, which mainly consist of ARDS but also arrhythmias, acute cardiac injury, and shock. A higher prevalence of such complications has also been reported in patients with comorbidities, such as systemic hypertension, coronary artery disease, diabetes mellitus, obesity, chronic obstructive lung disease (COPD), and asthma.^(5-9)^ The membrane-bound angiotensin-converting-enzyme 2 functions as a receptor for SARS-CoV-2, which partially explains some of these risk factors.^(10)^ However, the related epidemiology, clinical course, pathogenesis, and risk factors for complications of COVID-19 are not yet completely understood.

A better understanding of the pandemic worldwide is critical for the establishment of preventive and therapeutic measures. The aim of this study is to describe the clinical characteristics and risk factors for needing mechanical ventilation (MV) in a private hospital in Southern Brazil, which was one of the first hospitals assisting COVID-19 patients.

## METHODS

A retrospective cohort study was performed based on the review of electronic medical records of hospitalized patients from March 17, 2020 to May 3, 2020 at *Hospital Moinhos de Vento*, a private hospital in Porto Alegre, Brazil.

All hospitalized adult patients with confirmed infection due to SARS-CoV-2 were included in the study. The diagnosis was confirmed by reverse transcription-polymerase chain reaction (RT-PCR) collected through nasopharyngeal and/or oropharyngeal swab.

The need for MV was the primary outcome. Admission to the intensive care unit (ICU), ICU length of stay, duration of MV, and death were secondary outcomes analyzed in our study. We obtained epidemiological, demographic, clinical, treatment, and outcome data as well as admission laboratory and radiological data from the patients’ medical records. Further, an institutional routine of follow-up was implemented during the pandemic, consisting of a phone call for all patients hospitalized due to COVID-19 28 days (± 3 days) after discharge. During this contact, the need for new hospital admission and/or death was evaluated. Data of patients admitted during the study period obtained retrospectively through these follow-ups were included. Chest *computed tomography* (CT) results were classified by assistant radiologists as COVID-19 typical (peripheral bilateral or multifocal ground-glass opacities with or without consolidation or visible intralobular signs; reverse halo sign or other findings of organizing pneumonia), indeterminate or atypical (if there were abnormalities but without a typical pattern) or normal. Chest CTs were evaluated as a risk factor for our main outcome comparing abnormalities in < 50% *versus* ≥ 50% of the lung parenchyma.

Regarding the statistical analysis, continuous and categorical variables were presented as the medians, interquartile range (IQR), and n (%), respectively. The Shapiro-Wilk test of normality was used, and a two-sided α of less than 0.05 was considered statistically significant. For bivariate analysis, Pearson’s Chi-square test was used for categorical variables. The odds ratio was estimated through multivariable logistic regression. Models were built in a hierarchical sequence based on the literature review, starting with age and gender (model 1); adding obesity and comorbidities (model 2); and in the last level, adding CT results, C-reactive protein, lymphocytes and D-dimers (model 3). A sensitivity analysis was performed with three different cut-off levels of D-dimers (500ng/mL, 1,500ng/mL and 3,000ng/mL). Statistical analyses were performed using SAS software (Statistical Analysis System, SAS Institute Inc., Cary, N.C.) version 9.4.

The study was approved by the Institutional Review Board of *Hospital Moinhos de Vento* (protocol 30749720.4.1001.5330). Informed consent was waived due to the retrospective design of the study. Confidentiality regarding patients’ identity was assured.

## RESULTS

From March 17th, 2020 to May 3rd, 2020, 88 (60.27%) patients were hospitalized at *Hospital Moinhos de Vento*, 29 (32.9%) were admitted to the ICU and 18 (20.5%) needed invasive MV. Nine patients (10.2%) died during the first hospitalization. During the follow-up, in which all survivors were contacted, 7 were hospitalized within 28 days of discharge, with one of these patients dying due to abdominal sepsis.

Most patients (65.9%) reported three or more symptoms, three-quarters reported fever and 71.6% reported cough. Approximately 20% of patients presented with oxygen saturation of less than 93% at the first emergency assessment. In table 1, characteristics of the patients are shown according to the need for MV. Most of them were male (67.1%) and less than 65 years of age (56.8%). The median age was 63 years, ranging from 30 to 92 years (IQR 49 - 71). Regarding comorbidities, 25.8% were obese, and approximately half referenced two or more comorbidities. Abnormal counts of D-dimers (≥ 500ng/mL) and/or C-reactive protein (> 5mg/L) occurred in 43 (53.75%) and 39 (45.35%) patients, respectively. Seventy-nine patients (91.86%) had abnormal chest CT. The parameters of MV are summarized in table 2.

**Table 1:** Demographic, socioeconomic and clinical characteristics

Characteristics	MV	No MV	p value
Sex			0.60
Female	5 (27.8)	24 (34.3)	
Male	13 (72.2)	46 (65.7)	
Age ≥ 65 years	16 (88.8)	22 (31.4)	< 0.01
Comorbidities			
Obesity,	3 (16.7)	20 (28.6)	0.31
Hypertension,	11 (61.1)	25 (35.7)	0.05
Diabetes	4 (22.2)	13 (18.6)	0.72
Chronic pulmonary disease (asthma and COPD)	8 (44.4)	8 (11.4)	< 0.01
Coronary artery disease,	2 (2.3)	3 (4.3)	0.27
Cancer	1 (5.56)	4 (5.7)	0.98
Number of comorbidities			
None	1 (5.6)	20 (28,6)	0.04
One	4 (22.2)	21 (30.0)	
At least two	13 (72.2)	29 (41.4)	
D-Dimers	903.5 [561.0 - 1730.0]	470.5 [296.0 - 693.0]	< 0.01
D-Dimers (≥ 500ng/mL)	15 (83.3)	28 (45.2)	< 0.01
C-reactive protein	10.8 [3.2 - 14.4]	4.1 [2.1 - 8.9]	0.10
C-reactive protein (> 5mg/L)	12 (70.6)	27 (39.1)	0.02
Lymphocytes	930.0 [590.0 - 1160.0]	1270.0 [920.0 - 1600.0]	0.02
Lymphocytes (< 900/mm3)	9 (50.0)	15 (21.7)	0.02
Chest computed tomography			< 0.01
< 50%	9 (50.0)	63 (95.5)	
≥ 50%	9 (50.0)	3 (4.5)	

MV - mechanical ventilation; COPD - chronic obstructive lung disease. Results expressed as n (%) or median [interquartile range].

**Table 2:** Mechanical ventilation parameters, laboratory and radiological

Mechanical ventilation parameters	
Invasive mechanical ventilation	18 (20)
Plateau pressure (cmH_2_O)	
Day 1	21 [19 - 24.75]
Day 2	23 [20.25 - 27.5]
Day 3	23 [20 - 26]
Driving pressure (cmH_2_O)	
Day 1	10 [9 - 12.5]
Day 2	11 [9.5 - 12]
Day 3	10 [9 - 12.5]
PEEP (cmH2O)	
Day 1	10 [9.5 - 12.5]
Day 2	10 [8.75 - 12.5]
Day 3	10 [8 - 14]
Pulmonary compliance (mL/cmH_2_O)	
Day 1	40 [37.5 - 51.66]
Day 2	45.45 [35.83 - 47.33]
Day 3	45.83 [35.92 - 48.44]
Laboratory test	
PaO_2_/FiO_2_ after intubation (mmHg)	
Day 1	257 [196 - 359]
Day 2	346.66 [199 - 405]
Day 3	281.25 [202 - 358]
PaO_2_/FiO_2_ < 100mmHg	0
PaO_2_/FiO_2_ 100 - 199mmHg	7 (38.88)
PaO_2_/FiO_2_ 200 - 299mmHg	7 (38.88)
PaO_2_/FiO_2_ > 299mmHg	4 (22.22)

PaO_2_ - partial pressure of oxygen; FiO_2_ -fraction of inspired oxygen. Results expressed as n (%) or median [interquartile range].

The median length of stay was 9 days (5 - 48.5), the median length of ICU stay was 23 days (4 - 38) and the median length of MV was 29.5 days (18 - 45). The median duration of MV among patients who died and survivors was 36 (24 - 49) and 20 (18 - 37) days, respectively. During the first hospitalization, 89.9% and 96.6% of patients received hydroxychloroquine and azithromycin, respectively.

In bivariate analysis, age ≥ 65 years, presence of comorbidities, D-dimers ≥ 500 ng/mL, C-reactive protein > 5mg/L, and lymphocytes < 900 cells/mm^3^ and chest CT abnormalities in more than 50% of the lung parenchyma were associated with higher odds of needing MV. After multivariable adjusting, only age ≥ 65 years was associated with the outcome, odds ratio (OR) 8.4 (95% confidence interval - 95%CI 1.3 - 55.6), as shown in table 3. A sensitivity analysis considering different cut-off levels of D-dimers was performed. Levels ≥ 1500ng/mL were associated with the need for MV in the bivariate analysis but not the adjusted analysis (p < 0.01 and 0.38, respectively), while levels ≥ 3000ng/mL were not associated with this outcome in either analysis (p = 0.09 and 0.67, respectively).

**Table 3:** Logistic regression for factors predicting mechanical ventilation and adjusted analyses

	Unadjusted Odds ratio (95%CI)	p value	Model 1 Odds ratio (95%CI)	p value	Model 2 Odds ratio (95%CI)	p value	Model 3 Odds ratio (95%CI)	p value
Male sex	1.4 (0.4 - 4.3)	0.60	1.9 (0.5 - 6.3)	0.33	1.7 (0.5 - 6.0)	0.39	1.3 (0.3 - 5.5)	0.76
65 or more years	17.4 (3.7 - 82.6)	< 0.01	18.7 (4.0 - 86.5)	< 0.01	15.4 (3.6 - 65.5)	< 0.01	8.4 (1.3 - 55.6)	0.02
Obesity	0.5 (0.1 - 1.9)	0.31			1.0 (0.2 - 5.2)	0.96	1.6 (0.4 - 7.0)	0.53
One or more comorbidities	6.8 (0.8 - 54.6)	0.07			2.5 (0.3 - 19.9)	0.37	2.0 (0.3 - 13.1)	0.48
D-dimers ≥ 500 (ng/mL)	6.1 (1.6 - 23.1)	< 0.01					2.6 (0.6 - 11.1)	0.18
C-reactive protein > 5 (mg/L)	3.7 (1.2 - 11.8)	0.02					2.3 (0.6 - 9.6)	0.24
Lymphocytes < 900 (cells/mm^3^)	3.6 (1.2 - 10.7)	0.02					1.2 (0.2 - 6.2)	0.80
Chest CT ≥ 50%	21.0 (4.8 - 92.4)	< 0.01					3.9 (0.5 - 34.1)	0.21

## DISCUSSION

To the best of the authors’ knowledge, this is one of the first reports describing clinical and demographic data and evaluating risk factors for complications of COVID-19 in Brazil and Latin America. We found that older age was a major risk factor for needing MV.

Most of our patients were male (67%), similar to that reported by previous studies.^(11,12)^ The vast majority were Caucasians with a high educational level, which does not reflect most Brazilian sociodemographics. Regarding the patients’ age, the median was 63 years, similar to previous studies.^(11-14)^ During 2019, 96.9% of our admitted patients were Caucasian, which may be related to demographic, economic, and sociocultural factors. Some data are available about the relationship between COVID-19 and ethnic background, with some studies suggesting that nonwhite individuals could be at higher risk of death.^(5,7,15-17)^

Since the beginning of the COVID-19 pandemic, older age has been consistently associated with a higher risk of complications. The presence of comorbidities, especially systemic hypertension, diabetes mellitus, coronary artery disease, COPD, malignancy, and obesity, has been associated with a worse prognosis and death. However, many original studies and systematic reviews to date with incomplete data regarding outcomes and comorbidities did not adjust for confounders. Similar to our findings, in one study reviewing 191 patients during the beginning of the pandemic in China, older age, but no comorbidities, was associated with a higher risk of death after adjusting for confounders.^(6)^ In a systematic review assessing the impact of diabetes mellitus as a risk factor for complications of COVID-19, age, and systemic hypertension were identified as important confounders.^(18)^ Nonetheless, a posterior Chinese study including 1,590 patients showed that systemic hypertension (hazards ratio - HR) 1.5, 95%CI 1.0 - 2.3), diabetes mellitus (HR 1.5, 95%CI 1.0 - 2.4), COPD (HR 2.6, 95%CI 1.44 - 5.0), and malignancy (HR 3.5, 95%CI 1.6 - 7.6) were independently associated with ICU admission, MV, and death.^(19)^ These results may suggest that comorbidities have an impact on prognosis, but less so than older age. Our finding of age older than 65 years as the only predictor for needing MV is in line with this rationale, and a larger sample may be required to assess the impact of comorbidities.

The case fatality rate during the first hospitalization in our sample was 10.2%. Overall case fatality rates reported were approximately 2% and 3% but reached up to 21% among those hospitalized.^(16,20)^ A sample of 88 patients in a single center at the beginning of the pandemic may limit the external validity of our findings. Furthermore, many factors, such as local age pyramid and even BCG vaccination, could also have affected this outcome.^(21,22)^

The median duration of MV of 29.5 days reinforces the long duration of COVID-19 severe disease reported by previous studies.^(23)^ However, to the best of our knowledge, no previous study has reported a length of MV as long as our report, suggesting that the characteristics and quality of care of our hospital may have contributed to this finding. The long course of severe COVID-19 justifies the concern about a health system collapse in epidemiological scenarios of a rapid increase in the number of new cases as well as the global efforts to maintain social distancing.^(24)^

Regarding the chest CT findings, more than 91.86% of the hospitalized patients showed abnormalities, such as bilateral peripheral ground-glass opacities. In one of the first studies from Wuhan including 90 patients, similar findings were found in 72% of patients, while pleural and pericardial effusion and lymphadenopathy were uncommon, as in the present study.^(25)^ Ground-glass opacities were also demonstrated in 77% of patients in the cohort of Song et al., as well as consolidation and interlobular septal thickening in 59% and 75% of those individuals, respectively.^(26)^

This study has several limitations. First, the sample size may have underpowered the adjusted analysis of risk factors for the need for MV, particularly for risk factors less strongly associated with complications than older age. However, regardless of the statistical analysis, the description of clinical and demographic characteristics of one of the first Latin American cohorts has increased global knowledge about the pandemic. Moreover, the adjusted analysis allowed us to clarify that some potential risk factors identified in bivariate analysis were not associated with prognosis. Second, the number of deaths, the main outcome in many reports, limits the analysis of this outcome. However, the need for MV is one of the best markers of critical disease, particularly ARDS, and its clinical indications are less subject to bias. Third, due to the retrospective design, some variables, such as laboratory tests, may not have been collected systematically or may be available from different periods during the course of the disease. Nevertheless, most of our predictors are not subject to such bias. Fourth, a small case series from a single private not-for profit hospital is unlikely to reflect the large healthcare system of a country with continental size.

## CONCLUSION

This study provides original and early data of the COVID-19 pandemic in Brazil, providing insightful clinical data, such as patients’ characteristics and risk factors for COVID-19 related to complications in Brazilian patients. In terms of severity predictors, older age was the only independent variable associated with the need for mechanical ventilation. Further studies with larger sample sizes are needed to better understand COVID-19 and the risk factors for complications in a middle-income population setting, such as Brazil.
